# Deficiency in the *msbB* Gene Reduced the *Salmonella* Typhimurium Virulence Through Mechanisms Beyond LPS Modification

**DOI:** 10.3390/microorganisms13112510

**Published:** 2025-10-31

**Authors:** Ling Yang, Zhuodong Chai, Jiaqian Qi, Yan Zhang, Yuqi Zhou, Zhenyu Li, Yinan Wei

**Affiliations:** Department of Pharmaceutical Sciences, Irma Lerma Rangel School of Pharmacy, Texas A&M University, College Station, TX 77843, USA; ling.yang@tamu.edu (L.Y.); zdmaccc@tamu.edu (Z.C.); qijq@tamu.edu (J.Q.); yzh422@tamu.edu (Y.Z.); yuqi.zhou@tamu.edu (Y.Z.)

**Keywords:** *Salmonella* Typhimurium, *msbB*, TLR4, virulence factors, NLRC4 inflammasome

## Abstract

The *Salmonella enterica* serovar Typhimurium (ST) mutant lacking the *msbB* gene (Δ*msbB*) has been widely studied as a candidate for attenuated bacterial vectors in therapeutic applications. Deletion of *msbB* results in LPS with under-acylated lipid A, which lowers endotoxicity while maintaining structural integrity. This attenuation has traditionally been attributed to reduced TLR4 activation due to weaker interaction between the modified lipid A and TLR4. In our study, we confirmed that Δ*msbB* ST was less lethal than wild-type (WT) ST in a mouse sepsis model. However, this difference persisted even in TLR4- and caspase-11-deficient mice, suggesting that LPS signaling is not the primary determinant of virulence. In vitro, bone marrow–derived macrophages (BMDMs) from TLR4- or caspase-11-deficient mice showed only modest reductions in ST-induced cell death and cytokine production. Importantly, Δ*msbB* ST behaved similarly to WT ST in these assays, further indicating that LPS-mediated signaling is not central to the observed attenuation. Our previous studies showed that ST-induced mortality in mice is primarily mediated through NLRC4 activation. Using qPCR and immunoblotting, we found that expression of NLRC4 activators was diminished in the Δ*msbB* strain. Additionally, the mutant exhibited increased outer membrane permeability—likely contributing to its heightened antibiotic sensitivity—and reduced motility due to lower flagellin protein levels. In summary, the attenuation of virulence observed in the Δ*msbB* strain is not directly due to altered LPS–TLR4 interactions, but rather an indirect effect of diminished expression of virulence factors that activate the NLRC4 inflammasome.

## 1. Introduction

*Salmonella* is a genus of Gram-negative, non-spore-forming, rod-shaped bacteria and is among the most common pathogens responsible for foodborne illnesses [1]. The CDC estimates that *Salmonella* causes about 1.35 million illnesses and 420 deaths in the United States every year, with the most severe cases occurring in young children, the elderly, and immunocompromised individuals [2]. *Salmonella enterica* serovar Typhimurium (ST) invades and replicates within host epithelial cells, dendritic cells, and macrophages via multiple mechanisms, most of which are mediated by virulence factors encoded in highly conserved *Salmonella* Pathogenicity Islands (SPIs) [3,4]. These SPIs encode genes essential for bacterial virulence, such as those of the type III secretion system (T3SS) [5,6], which are potent activators of the NLRC4 inflammasome [7,8].

NLRC4 activation represents a rapid and specific immune response, enabling early elimination of invading pathogens. Compared to other inflammasomes like NLRP3, NLRC4 shows greater specificity due to its selective interactions with distinct NAIP receptors. In ST, known NLRC4 activators include the T3SS needle protein PrgI (recognized by NAIP1), the rod protein PrgJ (recognized by NAIP2), and flagellins FliC/FljB (recognized by NAIP5 and NAIP6) [9,10]. Once activated, the NLRC4 inflammasome not only promotes caspase-1 activation and the release of proinflammatory cytokines such as IL-1β but also amplifies the inflammatory response through the NF-κB signaling pathway [8]. While this response aids in pathogen clearance, excessive activation can result in tissue damage and organ failure. Our recent study showed that exposure to Gram-negative bacteria or flagellin leads to NLRC4-dependent lethal lung injury in mice [11].

The outer membrane lipopolysaccharide (LPS) of ST plays a key role in host–pathogen interactions. Modifications to the phosphate groups or the number of acyl chains on the lipid A moiety of LPS are important strategies by which the bacterium evades immune detection [12,13]. The *msbB* gene encodes an acyltransferase responsible for attaching a myristoyl group to the Kdo_2_–lipid IVA intermediate during lipid A biosynthesis [14]. Deletion of *msbB* results in LPS molecules with one fewer acyl chain in lipid A, significantly reducing the virulence of the strain [15,16]. The attenuated virulence has largely been attributed to decreased stimulation of the TLR4-mediated proinflammatory pathway, as under-acylated lipid A is less effective at activating TLR4 [15,16,17,18]. In murine models, Δ*msbB* ST exhibits markedly reduced pathogenicity, with no mortality or weight loss observed following oral administration of up to 10^9^ CFU per mouse [19]. This stands in sharp contrast to wild-type ST, which causes severe enterocolitis and systemic infection at doses as low as 10^5^ CFU [16]. Matsuura et al. demonstrated that the proinflammatory activity of LPS diminishes with a reduction in acyl chains, highlighting the critical role of lipid A acylation in immune activation [20]. Moreover, reduced proinflammatory activity of *msbB* mutant LPS has been observed in various Gram-negative bacteria, indicating a conserved mechanism [21,22,23]. While previous investigations into the Δ*msbB* mutant’s attenuation have focused on reduced LPS–TLR4 signaling, our recent observations suggest that ST virulence is primarily driven by NLRC4 inflammasome activation and subsequent cytokine release. Since TLR4 signaling is not directly involved in NLRC4 activation, we hypothesized that deletion of *msbB* leads to additional, indirect changes that contribute to virulence attenuation. In this study, we first employed a murine intraperitoneal sepsis model to demonstrate that neither TLR4 nor caspase-11—key sensors of LPS—are major contributors to the attenuation observed in Δ*msbB* ST. Next, using cultured bone marrow-derived macrophages (BMDMs), transcriptomic analysis, and biochemical assays, we identified additional mechanisms involved in the reduced virulence of the Δ*msbB* strain. Our findings suggest a multifactorial basis for attenuation, involving not only hypoactivation of TLR4 but also impaired inflammasome signaling and likely alterations in bacterial protein expression or secretion. These insights expand our understanding of how *msbB* deletion affects *Salmonella* virulence and underscore the complexity of host–pathogen interactions beyond LPS structure alone.

## 2. Methods and Materials

### 2.1. Mice

Wild-type (C57BL/6J) mice, along with TLR4^-/-^, Caspase-11^-/-^, and NLRC4^-/-^ knockout strains, were utilized in this study. All mice were housed under specific pathogen-free conditions at the Texas A&M University Animal Care Facility, in compliance with institutional guidelines and those established by the National Institutes of Health. All procedures were approved by the Texas A&M University Institutional Animal Care and Use Committee (IACUC). Both male and female mice, aged between 8 and 14 weeks, were included and gender-matched across experimental groups.

### 2.2. Bacteria

*Salmonella enterica* serovar Typhimurium (ATCC 14028) (WT) were obtained from the American Type Culture Collection (ATCC, Manassas, VA, USA). The *msbB* knockout strain (Δ*msbB*) was created using the Quick & Easy *E. coli* Gene Deletion Kit (Gene Bridges GmbH, Heidelberg, Germany) following manufacturer’s instruction. Freshly cultured bacteria were grown in LB broth at 37 °C with shaking until reaching the logarithmic phase, and their concentration was estimated by measuring optical density at 600 nm (OD_600nm_).

### 2.3. Isolation of Bone Marrow-Derived Macrophages (BMDMs)

BMDMs were generated from mouse bone marrow following established protocols [24]. In brief, bone marrow cells were harvested by flushing femurs and tibias with RPMI 1640 medium. The resulting suspension was filtered through a 70 μm cell strainer to remove debris and seeded into Petri dishes containing BMDM culture medium. The culture medium consisted of complete RPMI 1640 supplemented with 15% L929 cell-conditioned medium, 1% L-glutamine, 1% HEPES buffer, and 1% penicillin-streptomycin (100 U/mL and 100 μg/mL, respectively). Cells were incubated at 37 °C in a humidified 5% CO_2_ environment for 6–7 days. To eliminate non-adherent cells, the medium was replaced on day 3 or 4. Fully differentiated, adherent macrophages were collected and used for downstream experiments.

### 2.4. In Vitro Infection of BMDMs and LDH Assay

BMDMs were seeded in 96-well plates at a density of 1 × 10^5^ cells per well using BMDM culture medium. After overnight incubation at 37 °C with 5% CO_2_ to allow cell attachment, the medium was replaced with Opti-MEM (low-serum medium) following a PBS wash. Bacteria were resuspended in sterile PBS and added to the cells at a multiplicity of infection (MOI) of 25. To promote bacterial contact with the macrophages, plates were briefly centrifuged at 200× *g* for 5 min. After 90 min of infection, gentamicin (100 μg/mL) was added to kill extracellular bacteria and terminate the infection. Cytotoxicity was assessed by collecting 50 μL of supernatant and measuring lactate dehydrogenase (LDH) release using the CytoTox 96^®^ Non-Radioactive Cytotoxicity Assay kit (Promega, Madison, WI, USA), according to the manufacturer’s protocol.

### 2.5. Cytokine Quantification

For in vivo inflammation studies, C57BL/6J and the corresponding gene deficient mice *Tlr4*^-/-^, *Caspase-11*^-/-^, *Nlrc4*^-/-^ (8–14 weeks old, *n* = 4 per group) were administrated with 1 × 10^8^ CFU *Salmonella* in 0.2 mL sterile PBS intraperitoneally. Blood samples were collected via retro-orbital bleeding in an EDTA tube before injection or at selected time points (1.5 h, 4 h, and 6 h) following injection. Plasma was separated by centrifuging the blood sample at 10,000× *g* for 1 min at room temperature. IL-1β, IL-6, and TNFα levels in collected plasma were measured using ELISA kits (Invitrogen, Carlsbad, CA, USA) following manufacturer’s instructions.

### 2.6. Western Blot Analysis

BMDMs were seeded into 12-well plates at a density of 1 × 10^6^ per well and incubated overnight. The next day, culture supernatants were collected and subjected to trichloroacetic acid (TCA) precipitation to concentrate secreted proteins. For cellular protein extraction, cells were lysed directly in 1 × SDS loading buffer supplemented with 1% protease inhibitor cocktail (PI8340), followed by brief sonication. Protein samples were resolved by SDS-PAGE and transferred to PVDF membranes for immunoblotting. Pro-caspase-1 and caspase-1-p20 were detected using anti-caspase-1(p20) antibody (cat# AG-20B-0042, Adipogen, San Diego, CA, USA) at 1:1000 dilution. β-actin were detected using anti-β-actin antibody (cat# 4970, Cell signalling, Danvers, MA, USA) at 1:1000 dilution. Pro-IL-1β and IL-1β p17 were detected using anti-IL-1β antibody (cat# GTX74034, GeneTex, Irvine, CA, USA) at 1:1000 dilution. Flic were detected using anti-Flagellin Flic antibody (cat# mabg-flic-2, Invitrogen, San Diego, CA, USA) at 1:1000 dilution. PrgJ were detected by using *Salmonella* typhimurium PrgJ Polyclonal Antibody (Invitrogen, cat# PA5-117573). TolC were detected by using anti-TolC antibody sustained in our lab [25]. Anti-rabbit IgG (cat#BA1054, Boster, Pleasanton, CA, USA) and anti-mouse IgG (Boster, cat#BA1050) secondary antibody were applied at 1:15,000 and 1:3000 dilution, respectively. Blots were imaged using Azure^TM^ 500 imager (Azure biosytem, Dublin, CA, USA). Densitometric analysis of bands was carried out using ImageJ software (version 1.54p).

### 2.7. Prothrombin Time and Plasma Thrombin–Antithrombin Complex Measurement

Following previously established protocols with slight modifications [24], blood was collected from mice (*n* = 4–5 per group) anesthetized with tribromoethanol (Avertin) via cardiac puncture using a 23-gauge needle attached to a syringe preloaded with 3.8% trisodium citrate (final blood-to-anticoagulant ratio of 7:1). The samples were centrifuged at 1500× *g* for 15 min at 4 °C to isolate plasma. Prothrombin time (PT) was measured manually using Thromboplastin-D reagent (cat#100357, Pacific Hemostasis, Waltham, MA, USA) in CHRONO-LOG #367 plastic cuvettes, following the manufacturer’s instructions. Plasma levels of thrombin–antithrombin (TAT) complexes were quantified using a mouse-specific ELISA kit (cat#ab137994, Abcam, Waltham, MA, USA), with plasma samples diluted 1:50, as per the supplier’s guidelines.

### 2.8. RNA Extraction and RT-qPCR

Total RNA was isolated from wild type ST and its isogenic Δ*msbB* mutant. Bacterial pellets were collected and resuspended in Trizol reagent (Invitrogen, Carlsbad, CA, USA) for RNA extraction. The resulting RNA pellet was air-dried and resuspended in RNase-free water. RNA purity and concentration were measured using a Cytation5 microplate reader (BioTek, Winooski, VT, USA). 500 ng of RNA were reverse transcribed using an iScript™ gDNA Clear cDNA Synthesis Kit (cat# 1725035, Bio-Rad, Hercules, CA, USA). Real-time PCR was performed using iTaq SYBR green supermix (Bio-Rad, 1725121) with detection on a CFX96 system (Bio-Rad). The primers used in the qPCR are listed below: for *fljB* (FljB-F:5′-TGACGCTACCGATGCTAATG-3′, FljB-R:5′-TCCTGTCGCTTCATCGTAATC-3′, this study); for flic (FliC-F:5′-GTCGCTGTTGACCCAGAATAA-3′, FliC-R:5′-CGTCTTTC GCGCTGTTGATA-3′, this study); for prgI (PrgI-F:5′-GTAACAGAGGGCGTGGATAAA-3′, PrgI-R: 5′-TTGCCGGTTACGGTACAA-3′); for *prgJ* (PrgJ-F: 5′-GGCGGTCAATATCAG GTCTATG-3′, PrgJ-R: 5′-GGTCCTCAATCCTGTTGGTAAT-3′, this study); for *16s rRNA* (16s rRNA-F: 5′-TACTGGAAACGGTGGCTAATAC-3′; 16s rRNA-R: 5′-TACCTCA CCAACAAGCTAATCC-3′, this study). Relative gene expression was calculated based on the 2^−ΔΔCT^ method, using 16S rRNA as the reference gene.

### 2.9. RNA Sequencing and Data Analysis

Total RNA was extracted from midlog-phase *S*. Typhimurium 14028 (WT) and its isogenic Δ*msbB* mutant (two independent colonies per genotype) as described above. After DNase I treatment, libraries were prepared with the Illumina TruSeq Stranded kit (LC sciences, Houston, TX, USA) and sequenced (2 × 150 bp) on a NovaSeq 6000 (Illumina, San Diego, CA, USA), yielding ≥ 20 million read pairs per sample.

Quality control (FastQC v0.11.9) and adaptor/low-quality trimming (Trimmomatic v0.39) preceded alignment to the *S.* Typhimurium 14028 reference genome (NC_016856.1) with HISAT2 v2.2.1. Gene-level counts were generated by featureCounts (Subread v2.0.3) using the NCBI annotation (GFF3). Differential expression between WT and Δ*msbB* was computed in R v4.3.1 with DESeq2 v1.38.0; genes exhibiting |log_2_(fold-change)| ≥ 1 and Benjamini–Hochberg–adjusted *p* < 0.05 were considered significant and visualized in a volcano plot. Significantly regulated genes were subjected to pathway over-representation analysis against the KEGG database using clusterProfiler v4.8.1 (hypergeometric test, *q* < 0.10). Enriched pathways—including “Flagellar assembly”, “Two-component system” and “Bacterial chemotaxis”—are displayed in Figure S2. All scripts are available upon request. Raw FASTQ files and the corresponding count matrix have been deposited in the NCBI Gene Expression Omnibus (accession GSE304712; the accession will be public upon acceptance).

### 2.10. Nitrocefin Hydrolysis Assay

Hydrolysis of nitrocefin by β-lactamase was used to monitor outer membrane permeabilization [26]. WT and Δ*msbB* ST were transformed with the plasmid pUC18, which encodes the periplasmic β-lactamase enzyme. Overnight cultures of the wild-type and pUC18-transformed bacteria were grown in LB broth at 37 °C with shaking. The next day, cultures were diluted 1:100 into fresh LB medium containing ampicillin (100 µg/mL) and grown to OD_600_~0.5. Cells were harvested by centrifugation at 4000× *g* for 10 min at room temperature, washed twice with 50 mM sodium phosphate buffer (PBS, pH 7.0), and resuspended to an OD_600_ of 1.0 in PBS. Cells were added to the 96-well plate at a final OD_600_ of 0.1. Nitrocefin was added to the well with a final concentration of 100 μM. Absorbance at 486 nm was recorded every 1 min for 10 min immediately after the addition of nitrocefin.

### 2.11. 1-N-Phenylnaphthylamine (NPN) Uptake Assay

NPN uptake assay was performed following the previous published methods with some modifications [27]. Briefly, bacterial cells were harvested from overnight cultures, washed, and resuspended in 5 mM HEPES buffer (pH 7.2) to an OD_600_~0.5. The assay was assembled in triplicate in a black 96-well plate with the following conditions: (a) Cells only: 100 µL of bacterial suspension HEPES buffer mixed with 100 µL of HEPES buffer; (b) NPN only: 195 µL of HEPES buffer mixed with 5 µL of NPN working solution dissolved in acetone (final NPN concentration = 10 µM); (c) Cells + NPN: 100 µL of bacterial suspension mixed with 95 µL of HEPES buffer and 5 µL of NPN working solution (final NPN concentration = 10 µM). Fluorescence was measured immediately after mixing using a microplate reader with excitation and emission wavelengths set at 355 nm and 405 nm, respectively. Fluorescence readings were recorded every 2 min for a total duration of 20 min.

### 2.12. Gram Staining

To prepare the slides, the bacterial overnight cultures were pelleted and resuspended in phosphate-buffer saline (PBS). A thin smear was prepared on a glass slide, air-dried, and heat-fixed. *Salmonella* cells were stained using a Gram Staining Kit (Thermo Scientific™, Waltham, MA, USA) according to the manufacturer’s instructions. After rinsing and drying, the stained cells were examined under oil immersion using a Keyence BZ Microscope ×800 (Keyence, Itasca, IL, USA).

### 2.13. Motility Assay

To assess the impact of *msbB* deficiency on bacterial motility, motility assays were conducted using both WT and Δ*msbB* ST strains. Bacterial cultures were grown overnight at 37 °C in 3 mL of LB broth with shaking at 250 rpm. Cells were harvested by centrifugation, washed once, and resuspended in sterile phosphate-buffered saline (PBS, pH 7.0) to an OD_600_ of 0.5. Motility was evaluated on 0.4% semi-solid agarose plates prepared in 10 cm polystyrene Petri dishes [28]. A sterile round filter paper disc (5 mm in diameter) was placed at the center of each agar plate, and 5 µL of bacterial suspension (OD_600_ = 0.5 in PBS) was spotted onto the disc. Plates were incubated at 37 °C for 20 h. Motility zones were then imaged and analyzed. Each assay was performed in three independent biological replicates.

### 2.14. Statistical Analysis

All data are presented as mean ± standard deviation (SD). Each group was tested in triplicate, and all experiments were performed independently at least three times. Statistical significance was assessed using multiple unpaired *t*-tests, as appropriate. All analyses were performed using GraphPad Prism 10 software based on biological replicate samples. Details regarding statistical significance, including the meaning of asterisks and comparisons between groups, are provided in the corresponding figure legends.

## 3. Results

### 3.1. Reduced Virulence in ΔmsbB ST Is Not a Direct Result of Altered LPS Signaling

**Figure 1:**
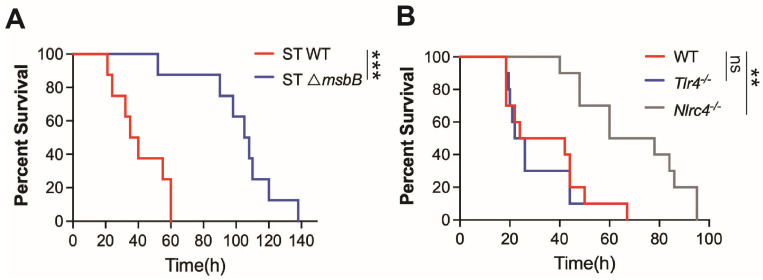
Mice survival. (**A**,**B**) C57BL/6J (WT), *Tlr4*^-/-^, *Nlrc4*^-/-^ and *Casp11*^-/-^ mice, 10–14 weeks old sex matched (*n*  =  8–10 per group), were intraperitoneally injected with 5 ×  10^6^ CFU bacteria (WT or Δ*msbB*) in 0.2 mL sterile saline. Kaplan–Meier survival curve for (**A**) WT vs. Δ*msbB* ST for WT mice, (**B**) WT vs. *TLR4*^-/-^ vs. *NLRC4*^-/-^ mice infected with WT ST were shown. ns: no significant difference, ** *p*  <  0.01, *** *p*  <  0.001, Log-rank (Mantel–Cox) test.

It has been generally accepted that the reduced virulence of the Δ*msbB* strain results from modifications in the LPS structure, which lead to attenuated TLR4-dependent inflammatory responses. However, our recent findings demonstrate that the NAIP/NLRC4 inflammasome, a LPS independent pathway, plays a critical role in the pathogenesis of ST infection [6]. Consistent with this, we observed that mice challenged with the Δ*msbB* strain exhibited significantly lower mortality rates compared to those infected with the parent strain (Figure 1A). As previously reported, WT ST induced similar mortality in both wild type and TLR4 deficient mice, whereas the deletion of the NLRC4 gene significantly prolonged survival (Figure 1B). In contrast, caspase-11 deficiency, which impairs intracellular sensing of LPS, provided only modest protection against ST infection [6].

**Figure 2:**
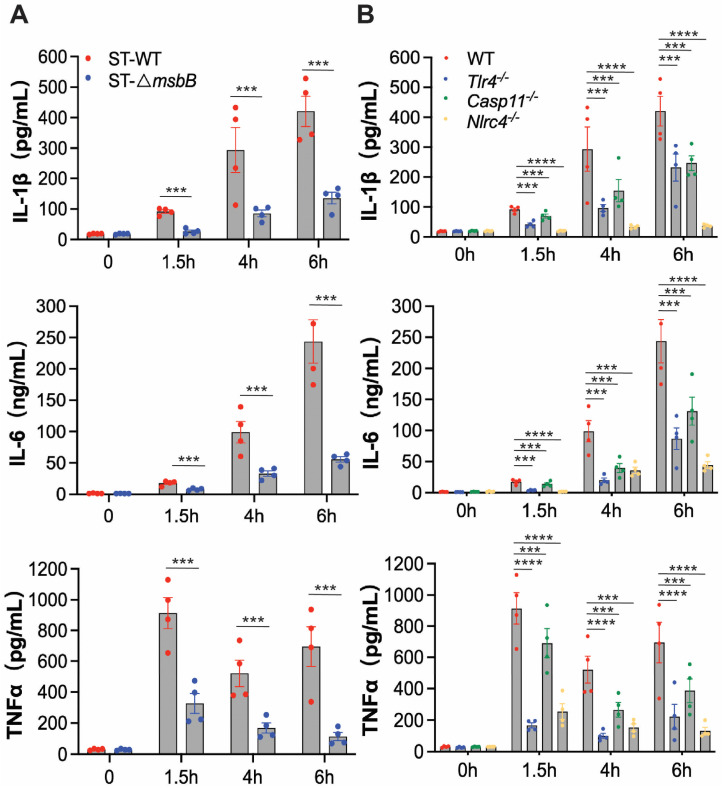
In vivo cytokine production by mice infected by *Salmonella*. (**A**,**B**) C57BL/6J WT or gene deficient mice (*n* = 4 per group) were intraperitoneally injected with 1 × 10^8^ CFU bacteria, and blood was collected at the indicated times through retroorbital bleeding. Plasma levels of IL-1β, IL-6 and TNFα were measured by ELISA. (**A**) Plasma levels of IL-1β, IL-6 and TNFα in WT mice infected with WT or Δ*msbB* ST. (**B**) Plasma levels of IL-1β, IL-6 and TNFα in WT, *Tlr4*^-/-^, *Casp11*^-/-^ and *Nlrc4*^-/-^ mice infected with WT. All data are presented as mean  ±  SEM (*n*  =  4 per group). *** *p*  <  0.001, **** *p*  <  0.0001, by using two-way ANOVA with Holm–Sidak multiple comparison test.

To further investigate the inflammatory response, we examined plasma levels of IL-1β, IL-6, and TNFα in mice following infection with either wild-type or Δ*msbB* ST (Figure 2A). Wild type mice were injected intraperitoneally with ST or the mutant Δ*msbB* ST, and blood was collected immediately before, or at 1.5, 4, or 6 h post infection. In plasma samples collected from mice treated with the parent ST strain, IL-1β and IL-6 levels progressively increased over time, while TNFα peaked at 1.5 h and decreased by approximately 30% at later time points. Although Δ*msbB* ST elicited time-dependent increases in the plasma levels of IL-1β, IL-6, and TNFα, the peak concentrations were significantly lower, decreased by approximately 70% to 80%, than those observed in mice infected with the parent strain.

To determine whether ST-induced inflammation is primarily driven by LPS, we measured plasma cytokine levels in wild type or *Tlr4*^-/-^, *Casp11*^-/-^, *Nlrc4*^-/-^ mice injected intraperitoneally with ST similarly as described above. Deletion of NLRC4 completely abolished IL-1β secretion and significantly reduced the secretion of the other two cytokines (Figure 2B). TLR4 deletion resulted in partial reduction in all three cytokines, although the effect was less pronounced than that observed in NLRC4 deficient mice. Similarly, caspase-11 deficiency led to partial decreases in cytokine levels, with effects on IL-1β and IL-6 comparable to those seen in *Tlr4*^-/-^ mice, but a more modest reduction in TNFα secretion. These results indicate that the NLRC4-dependent pathway plays a dominant role in ST-induced inflammation, while the LPS-mediated mechanisms, including those involving TLR4 and caspase-11, contribute to a lesser extent. This conclusion aligns with our previous findings highlighting the critical role of the NAIP/NLRC4 pathway in driving ST-induced inflammation and mortality.

### 3.2. ΔmsbB ST-Induces Less Pyroptosis of Macrophage

**Figure 3:**
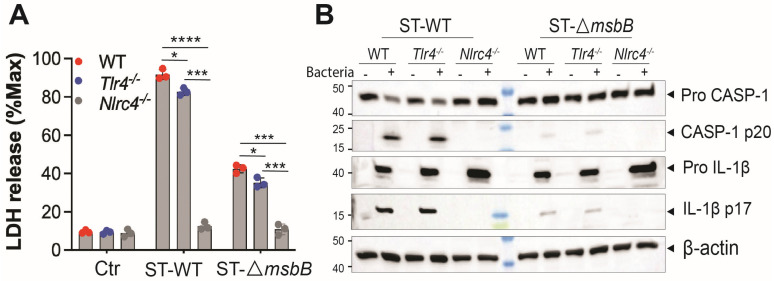
Δ*msbB* ST induced less pyroptosis in BMDM. (**A**) LDH release determined in the supernatant of different genotypes of BMDMs (5 × 10^5^ cells) after incubation with 25 MOI *Salmonella* for 90 min. (**B**) Representative image of immunoblotting analysis of caspase-1(full-length and p20) and IL-1β (full length and p17) in whole cell lysate of different strains of BMDMs after incubation with 25 MOI *Salmonella* for 90 min. * *p*  <  0.05, *** *p*  <  0.001, **** *p*  <  0.0001, analyzed using two-way ANOVA with Holm–Sidak multiple comparison test.

ST potently induces pyroptosis, a form of programmed cell death that contributes to the pathogenesis of ST infections [6]. ST-induced pyroptosis is dependent on the NAIP/NLRC4 inflammasome, as ST-triggered cell death in mouse bone marrow-derived macrophages (BMDMs) was completely abolished in both *Nlrc4*^-/-^ cells (Figure 3A) and *Naip*^-/-^ cells [6]. TLR4 deficiency had only a modest but statistically significant reduction in ST-induced cell death compared with wild-type BMDMs. Notably, infection with the Δ*msbB* strain resulted in significantly reduced cell death in BMDMs derived from both the parent mice strain and the two knockout strains.

Since ST induced pyroptosis requires caspase-1, we hypothesized that caspase-1 activation would be diminished in BMDMs infected with the Δ*msbB* strain. Indeed, Δ*msbB* induced significantly lower levels of caspase-1 cleavage and pro–IL-1β processing compared with the WT strain. (Figure 3B and Figure S1). The impact of TLR4 or NLRC4 knockout on ST induced caspase-1 and pro-IL-1β processing was also examined. While TLR4 deficiency had little effect, NLRC4 deletion completely abolished caspase-1 activation and pro-IL-1β processing in response to both wild-type and Δ*msbB* ST.

### 3.3. ΔmsbB ST Induces Less Coagulation in Mice

**Figure 4:**
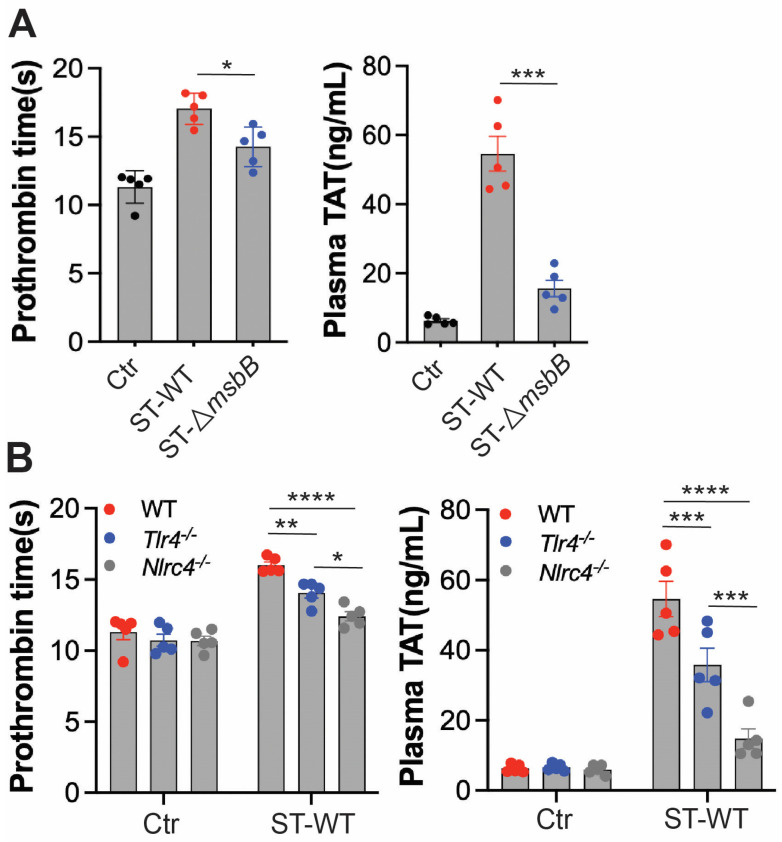
Δ*msbB* ST induced less coagulation than WT ST in mice. Measurement of prothrombin time and plasma TAT concentration in the indicated mice strain (*n*  =  5, 10–14 weeks old). (**A**) WT ST or Δ*msbB* ST injected into wild type mice. (**B**) WT ST injected into the indicated strain of mice. 1 × 10^8^ CFU *Salmonella* were injected intraperitonially. Blood was collected 8 h after injection, from control and infected mice through cardiac puncture into sodium citrate tube. Plasma was isolated for PT and TAT measurement. * *p*  <  0.05, ** *p*  <  0.01, *** *p*  <  0.001, **** *p*  <  0.0001, analyzed using two-way ANOVA with Holm–Sidak multiple comparison test. The black dots are data points (*n* = 5 per group) in control group.

Disseminated intravascular coagulation (DIC) is a well-established and often fatal complication of systemic bacterial infections. The presence of DIC markedly increases mortality in septic patients [29,30,31,32]. In previous studies, we observed that intraperitoneal injection of ST induces DIC in mice [6]. To determine whether deletion of the *msbB* gene reduces DIC severity, we assessed markers of coagulation activation in mice challenged with either wild-type ST or Δ*msbB* (Figure 4A). Prothrombin time (PT) was significantly prolonged following *Salmonella* infection. Likewise, plasma levels of the thrombin–antithrombin (TAT) complex were markedly elevated. Both indicators of coagulopathy were significantly attenuated in mice infected with Δ*msbB* compared to those infected with the wild-type strain.

We next examined the roles of TLR4 and NLRC4 in ST-induced DIC (Figure 4B). Deletion of either gene significantly reduced DIC severity, with NLRC4 deletion having a more pronounced effect than TLR4 deletion. These findings are consistent with the reduced inflammatory response and extended survival observed in NLRC4-deficient mice compared to TLR4-deficient mice.

Together, these results suggest that the protection against DIC conferred by *msbB* gene deletion is primarily due to reduced activation of the NLRC4 pathway, rather than diminished LPS/TLR4 signaling.

### 3.4. Deletion of msbB Leads to Reduced Expression of FliC/FljB and T3SS Effectors

**Figure 5:**
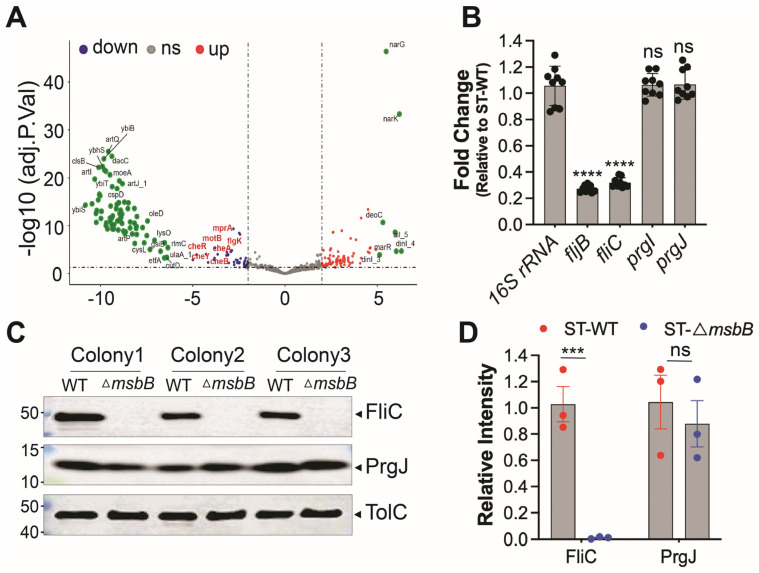
Reduced expression of FliC/FljB was observed in Δ*msbB* ST. (**A**) The volcano plot highlights several genes impacted by the deletion of *msbB*, with two group of pathway-specific genes highlighted: Two-component system—*cheA*, *cheB*, *cheR*, *cheY*, and Flagellar assembly—*flgK*, *motA*, *motB*. All are down-regulated. (**B**) Fold change of fljB, *fliC*, *prgI* and *prgJ* mRNAs in Δ*msbB* relative to those genes in WT ST, measured by RT-qPCR. (**C**) Representative image of immunoblotting analysis of FliC, PrgJ and TolC in WT and Δ*msbB* ST. Three colonies of each strain were selected and used for WB. (**D**) Relative density quantification for FliC and PrgJ bands, normalized to the density of TolC. *** *p*  <  0.001, **** *p*  <  0.0001, ns: no significant difference. Two-way ANOVA with Holm–Sidak multiple comparison test.

To investigate how deletion of a gene involved in LPS biosynthesis might influence activation of the NAIP/NLRC4 inflammasome, we performed RNA-seq to compare gene expression profiles between wild-type and Δ*msbB* ST strains. We hypothesized that *msbB* deletion indirectly reduced the expression of NAIP/NLRC4 activators. Differentially expressed genes were identified and KEGG analysis were conducted (Figure 5A and Figure S2). Detailed list of transcripts affected by the deletion is listed in Supplementary Table S1. Aside from the expected absence of *msbB* transcripts in the mutant, we found that the expression of fljB, which encodes a well-characterized NAIP/NLRC4 activator flagellin, was notably decreased. In addition, the KEGG analysis revealed that several pathways are affected by the *msbB* gene disruption, notably those involved in two-component system and flagella assembly.

Next, we performed qPCR analysis to compare the transcription level of well-established NLRC4 inflammasome activators, including *fljB*. In addition, we tested FliC, another subunit protein of flagellin, and T3SS apparatus proteins PrgI (forms the needle) and PrgJ (forms the inner rod). The 16S rRNA was used as a normalization control. The qPCR results indicate that the transcriptions of the two proteins that make up the flagellin subunits were down-regulated in the Δ*msbB* strain, while that for the T3SS inner rod and needle proteins were not (Figure 5B).

To further confirm these findings at the protein level, we performed Western blot analysis to assess the expression of FliC and PrgJ (Figure 5C,D). Consistent with transcript-level data, FliC protein levels were significantly reduced in the Δ*msbB* strain, while PrgJ expression remained unchanged. The outer membrane protein TolC was used as a loading control.

### 3.5. Other Factors That Might Affect the Virulence of the ΔmsbB Strain

**Figure 6:**
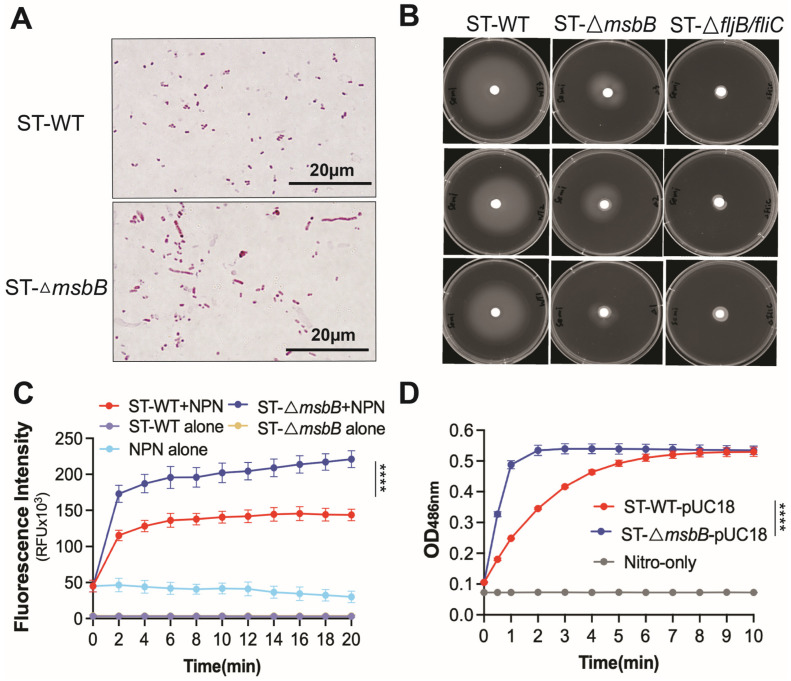
Comparison of morphology, motility and membrane permeability between WT and Δ*msbB* ST. (**A**) Bacteria morphology captured under oil immersion lense. Bacteria were treated with Gram staining. (**B**) Motility of WT and Δ*msbB* ST were measured on a semi-solid (0.4%) agar. Δ*fljB/flic* ST was used as the negative control. Cultures were incubated at 37 °C for 20 h. Plates are representative of 3 biological replicates. Membrane permeability analysis using NPN uptake assay (**C**) and nitrocefin hydrolysis assay (**D**) as described in Materials and Methods. **** *p*  <  0.0001, Two-way ANOVA with Holm–Sidak multiple comparison test.

Deletion of the *msbB* gene does not appear to affect bacterial growth in LB medium. Both growth rate and final cell density were comparable between the wild-type and Δ*msbB* strains (Figure S3A). However, when cultured on agarose plates, the Δ*msbB* strain tended to form elongated filaments, suggesting a defect in cell division (Figure 6A).

Earlier studies have also shown that the mutant is more sensitive to bile salts [14]. We next assessed the antibiotic sensitivity of the wild-type and mutant strains. The Δ*msbB* strain exhibited increased susceptibility to a range of antimicrobials spanning multiple classes and mechanisms, including quinolones, tetracycline, β-lactams, vancomycin, and polymyxin (Table 1). This observation prompted us to investigate whether the heightened sensitivity was due to increased membrane permeability. To address this, we performed 1-N-phenyl-1-naphthylamine (NPN) uptake assay and nitrocefin membrane leakage assay (Figure 6C,D). The hydrophobic dye NPN fluoresces intensely upon binding to the inner phospholipid bilayer of Gram-negative bacteria. When the outer membrane remains intact, it restricts NPN penetration and yields low fluorescence. In contrast, outer membrane leakage allows NPN to enter and results in markedly increased fluorescence. The fluorescence of the sample containing Δ*msbB* ST was significantly higher than the sample containing WT ST (Figure 6C), which indicates the loss of outer membrane integrity in the Δ*msbB* strain. Nitrocefin undergoes a color change from yellow to red upon hydrolysis by β-lactamases located in the periplasm of *Salmonella*. In strains with a leaky outer membrane, nitrocefin gains better access to the periplasmic space, resulting in more rapid hydrolysis. The Δ*msbB* strain showed significantly faster nitrocefin hydrolysis (Figure 6D), indicating increased outer membrane leakage caused by *msbB* deletion.

Finally, using qPCR and immunoblotting data, we confirmed that flagellin production is reduced in the Δ*msbB* strain. Given the importance of flagella for *Salmonella* motility, we conducted a motility assay (Figure 6B and Figure S3B). The Δ*msbB* strain exhibited impaired motility compared to the wild type. As a negative control, we included a flagellin-deficient strain (Δ*fljB/fliC*), which—as expected—showed minimal motility.

## 4. Discussion

The *msbB* gene is highly conserved among various Gram-negative bacteria, where it plays a critical role in the late-stage acylation of lipid A. This conserved function makes *msbB* an attractive target for attenuation strategies, as its deletion reduces inflammation while preserving antigen presentation when used in vaccine development. By delivering autoantigens and immunoregulatory cytokines with reduced endotoxicity, Δ*msbB*-based therapies have been developed to prevent and reverse of type 1 diabetes in NOD mice, comparable to previously validated attenuated strains but with improved safety and infection efficiency [15,33]. In addition, *msbB*-deficient ST strains have been investigated in cancer immunotherapy, where they tend to induce a Th2-skewed immune response, characterized by reduced IFN-γ and TNF-α production [34]. Since the balance between Th1 and Th2 responses is critical for chronic infection control and immune modulation, this Th2 shift likely contributes to improved safety and a lower risk of sepsis [34,35]. Importantly, unlike other LPS-detoxified mutants, *msbB* deletion does not significantly impair bacterial resistance to complement-mediated lysis or other innate immune mechanisms [36]. Moreover, Δ*msbB* strains retain tumor-targeting properties and exhibit partial suppression of tumor growth, supporting their utility as tumor-targeting bacterial vectors [37].

Given their potential as a delivery vesicle for various applications, it is essential to understand the mechanism underlying the reduced virulence of Δ*msbB Salmonella*. We propose that this attenuation extends beyond its canonical role in LPS modification. In this study, we demonstrate that deletion of *msbB* modulates the activation of the NLRC4 inflammasome, likely by altering the expression of proteins that act as NLRC4 activators. Infections with wild-type or Δ*msbB* strains in TLR4- or caspase-11-deficient mice led to similar mortality rates and partial reduction in cytokine profiles. In contrast, NLRC4 deficient mice were substantially protected, highlighting a critical role for this inflammasome in host response. NLRC4 is essential for immune responses against pathogens expressing type III secretion systems (T3SS) or flagellin, such as *Legionella pneumophila*, *Pseudomonas aeruginosa*, and *Shigella flexneri* [38,39]. In mice, intestinal epithelial cells (IECs) are a primary site of *Salmonella* infection, and epithelial-intrinsic expression of NAIP or NLRC4 is both necessary and sufficient to restrict bacterial burden, indicating a central role for the NAIP/NLRC4 inflammasome in mucosal immunity [40,41].

Consistent with our findings that ST induces pyroptosis in murine macrophages, recent studies have confirmed NLRC4 activation in human macrophages upon ST infection [10,42]. The magnitude of this activation is influenced by infection route, bacterial dose, and tissue tropism. We further show that Δ*msbB* attenuates *Salmonella*-induced pyroptosis in macrophages, a process mediated by NLRC4, suggesting that *msbB* deletion may interfere with the availability or recognition of NAIP/NLRC4 agonists through altered expression or secretion of virulence-associated factors.

Coagulation is a major contributor to host mortality following inflammasome activation. Previously, we demonstrated that activation of either the canonical inflammasome by the T3SS rod protein of Gram-negative bacteria, or the non-canonical inflammasome by LPS, can trigger systemic coagulation and widespread thrombosis [43]. Here, we extend these findings by showing that infection with ST resulted in prolonged PT and elevated TAT complexes in TLR4-deficient mice, though to a lesser level compared to those observed in C57BL/6 mice. In contrast, NLRC4-deficient mice displayed significantly reduced DIC markers. A similar reduction was observed during infection with the Δ*msbB* mutant, suggesting that ST-induced coagulation is primarily mediated through NLRC4, and that *msbB* deletion dampens this signaling. Supporting this, recent studies have identified gasdermin D-mediated pyroptosis as a drive of tissue-factor exposure and systemic coagulation downstream of inflammasome activation [44,45]. These findings help bridge the gap in understanding the link between NLRC4 inflammasome activation and coagulopathy, and suggest that Δ*msbB* may have broader therapeutic potential in mitigating lethal inflammatory complications.

Changes in LPS structure can also affect the overall composition and permeability of the bacterial envelope, influencing the exposure of other surface components such as lipoproteins or peptidoglycans. Grassl et al. showed that infection with Δ*msbB* ST triggered elevated inflammation in Nod1^-/-^ and Nod2^-/-^ mice, a phenotype linked to the loss of NOD1/2-mediated suppression of TLR2 signaling [16]. This may result from structural changes in LPS that increase the exposure of outer membrane lipoproteins recognized by TLR2. In our study, qPCR analysis revealed a marked downregulation of *fliC*, *fljB*, and other flagellar-associated genes, consistent with the observed reduction in NLRC4 inflammasome activation. This pattern aligns with findings in *Vibrio parahaemolyticus* Δ*msbB* mutant, in which Che et al. reported significant downregulation of multiple flagellar and impairing swarming motility [46]. Similarly, in a previous study, mutation of *msbB* (also known as *waaN*) disrupted the secretion of virulence-associated proteins [47]. Together, these results indicate that *msbB* deletion affects not only LPS modification, but also the expression of genes involved in motility and immune evasion. The reduced mobility and increase membrane permeability likely also contribute to the reduced virulence of the Δ*msbB* strain.

Although the mechanism directly linking *msbB* deletion to reduced flagellar gene expression remains unclear, it is plausible that changes in lipid A acylation or outer membrane fluidity influence the activity of two-component regulatory systems, which govern motility and virulence in *Salmonella* and other bacteria [48,49,50,51]. These envelope stress–responsive systems, including PhoP/PhoQ and PmrA/PmrB, are known to regulate genes involved in lipid A remodeling and resistance to antimicrobial peptides. Activation of the PhoP/PhoQ pathway has been reported to repress flagellar gene expression through the regulation of flhDC, the master flagellar operon [52]. Similarly, PmrA/PmrB activation can influence the expression of motility-related genes as part of a broader adaptive response to membrane stress [53]. Therefore, the altered lipid A acylation in the *msbB* mutant could indirectly lead to downregulation of flagellar genes via these regulatory networks, contributing to the observed attenuation in motility and virulence.

The formation of elongated, filamentous cells in the *msbB* mutant may represent a stress response to altered outer membrane architecture resulting from under-acylated lipid A. Proper lipid A acylation is critical for maintaining outer membrane stability and for efficient cell division. Perturbation of lipid A structure can lead to defects in septum formation and delayed cytokinesis, resulting in filamentation [54]. In Salmonella, filamentous morphology has been linked to impaired invasion of epithelial cells and reduced survival within macrophages [55,56]. Therefore, the filament formation observed in the *msbB* mutant likely reflects underlying envelope stress that compromises normal cell division and motility, contributing to its attenuated virulence phenotype.

Despite these insights, several limitations should be noted. Our experiments primarily used intraperitoneal infection models in vivo and macrophage assays in vitro, which may not fully reflect the dynamics of natural *Salmonella* infection, particularly those involving oral transmission and the intestinal mucosa. Future studies using oral infection models are necessary to determine whether NLRC4-dependent mechanisms similarly operate in mucosal environments. Additionally, the regulatory connection between lipid A acylation and virulence gene expression remains poorly defined. Addressing these questions will be key to fully elucidating the broader impact of *msbB* on host–pathogen interactions.

In summary, our study shows that the attenuation of Δ*msbB* ST is not solely due to reduced LPS-TLR4 signaling from modified LPS, but also involves suppression of NLRC4 inflammasome activation, downregulation of flagellar genes, altered outer membrane integrity, and reduced pyroptosis and coagulation responses. These findings highlight a broader immunomodulatory role for *msbB* and reinforce its potential as a target for developing safer bacterial vectors for therapeutic use. Further investigation of the links between lipid A structure to virulence gene regulation could reveal new strategies for microbial engineering and host-directed therapies.

## Figures and Tables

**Table 1:** Minimum Inhibitory Concentration (MIC, µg/mL) values of 26 selected antimicrobial drugs or compounds against WT and Δ*msbB* ST.

Antimicrobials	WT	Δ*msbB*	Antimicrobials	WT	Δ*msbB*
Quinolones/Fluoroquinolones					
Nalidixic acid	8	**2**	Pefloxacin	0.25	**0.03**
Cinoxacin	8	**1**	Moxifloxacin	0.06	**0.015**
Ofloxacin	0.12	0.06	Marbofloxacin	0.015	0.008
Fleroxacin	0.25	**0.06**	Sparfloxacin	0.015	**0.002**
Levofloxacin	0.03	**0.008**	Enrofloxacin	0.015	0.004
Ciprofloxacin	0.015	0.008	Lomefloxacin	0.12	**0.03**
**Penicillins/Cephalosporins**					
Ampicillin	0.5	0.25	(+)-6-Aminopenicillin acid	32	16
Ticarcillin	2	1	Cefuroxime	16	8
Carbenicillin	4	2	Cefotaxime	0.12	0.06
**Tetracyclines**					
Tetracycline	2	**0.5**	Chlortetracycline	2	**0.5**
Minocycline	2	**0.5**			
**Other types**					
Chloramphenicol	4	2	Vancomycin	640	**80**
Polymyxin B	8	**2**	Ethidium bromide	2048	**128**
Linezolid	256	**64**			

The bold font indicates the MIC difference between ST-WT and ST-△*msbB* is greater than 2 folds.

## Data Availability

The original data presented in the study are openly available in the NCBI Gene Expression Omnibus (accession GSE304712).

## Supplementary Materials

The following supporting information can be downloaded at: https://www.mdpi.com/article/10.3390/microorganisms13112510/s1, Figure S1: (A,B) Relative band intensity quantifications for caspase1-p20 (A) or IL-1β (B) bands, normalized to the band density of β-actin. All data are presented as mean ± SEM (*n* = 3 per group). **** *p*  <  0.0001, analyzed using two-way ANOVA with Holm-Sidak multiple comparison test; Figure S2: Pathways were identified in KEGG analysis as affected by the *msbB* knockout; Figure S3: (A) Growth curves (OD600) for WT and Δ*msbB* ST in LB broth. (B) Motility diameters of the area of bacteria growth in (Figure 6B). ** *p*  <  0.01, *** *p*  <  0.001, ns: no significant difference, Two-way ANOVA with Holm-Sidak multiple comparison test; Table S1: Gene with different expression in the *msbB* knockout strain.
