# Supplementary material for: Deficiency in the msbB Gene Reduced the Salmonella Typhimurium Virulence Through Mechanisms Beyond LPS Modification
Source: Microorganisms. 2025 Oct 31;13(11):2510. doi: 10.3390/microorganisms13112510 (PMC12654218; doi:10.3390/microorganisms13112510)
Supplement: Supplementary file 1 [file microorganisms-13-02510-s001.zip › microorganisms-3870382-supplementary.pdf]

Supplementary Data

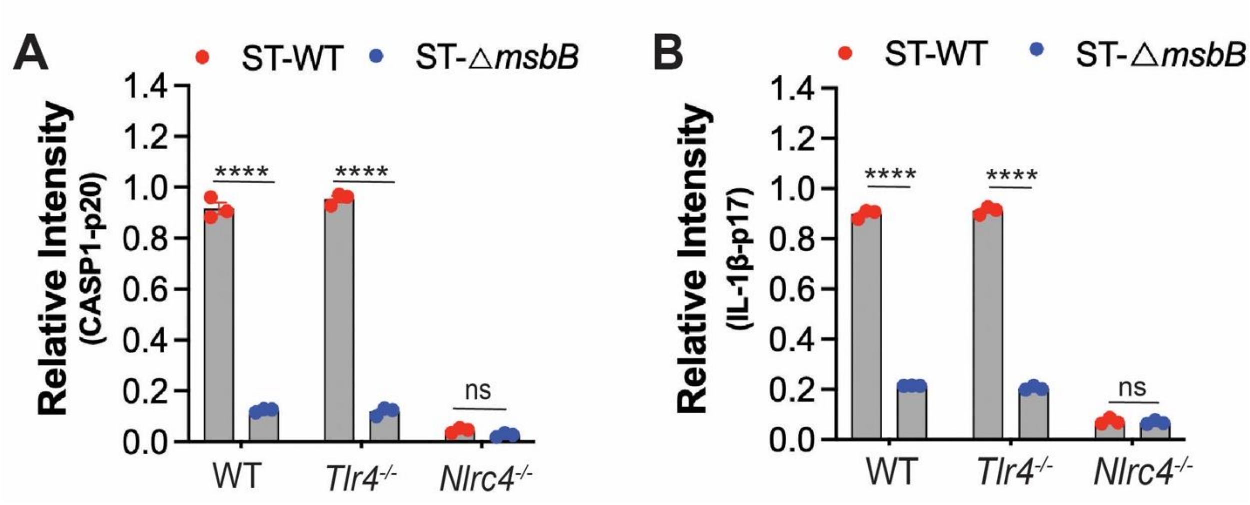

Figure S1. (A-B) Relative band intensity quantifications for caspase1-p20 (**A**) or IL-1β (**B**) bands, normalized to the band density of β-actin. All data are presented as mean ± SEM (n = 3 per group). ****$p < 0.0001$, analyzed using two-way ANOVA with Holm-Sidak multiple comparison test.

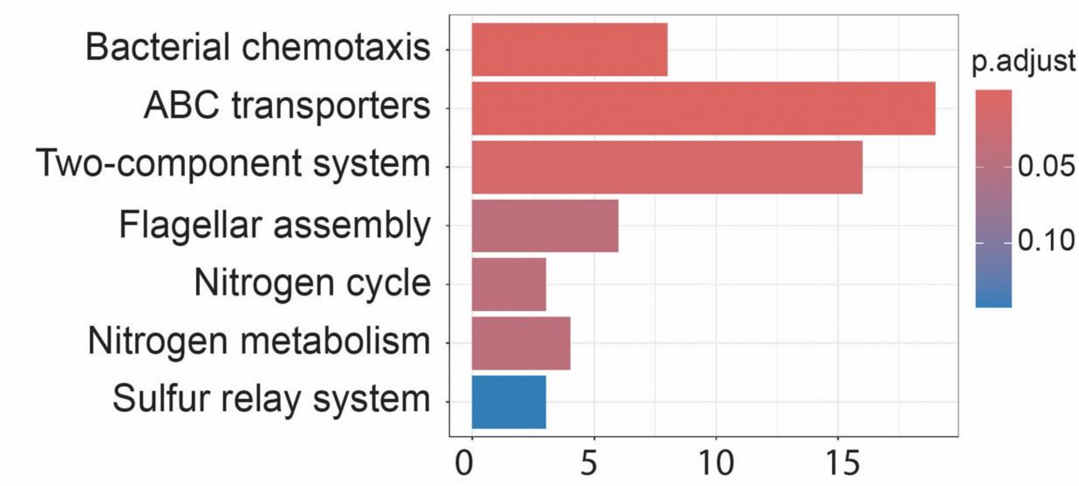

Figure S2. Pathways were identified in KEGG analysis as affected by the *msbB* knockout.

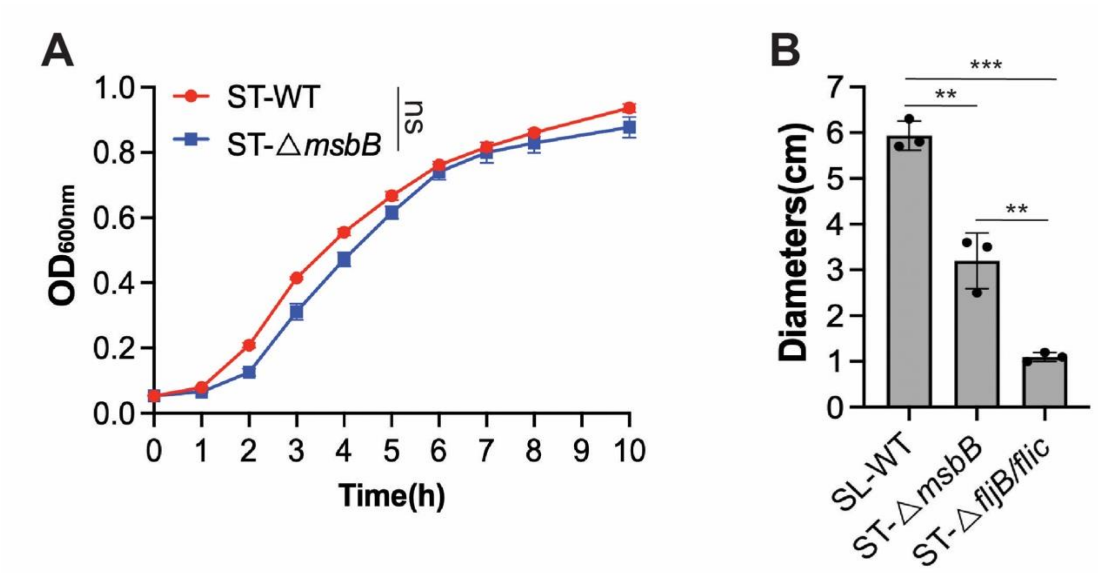

Figure S3. (**A**) Growth curves (OD600) for WT and *ΔmsbB* ST in LB broth. (**B**) Motility diameters of the area of bacteria growth in (Fig.6B). **$p < 0.01$, ***$p < 0.001$, nsno significant difference, Two-way ANOVA with Holm-Sidak multiple comparison test.

Table S1. Gene with different expression in the *msbB* knockout strain.

	gene_id	AveExpr	logFC	lfcSE	P.Value	adj.P.Val	gene
1	ENSB:-5dV6Ix6VxDTrdR	294.6714	3.44246	1.424834	0.000145	0.003247	dinI_1
2	ENSB:-Jq2edtTvDsL_nO	21.47359	-6.74632	1.52362	5.59621454846814e-07	2.16451846187707e-05	mntR
3	ENSB:-kcQNjBy7spd4Di	1168.267	-8.92609	1.232526	1.30614323060616e-13	1.05631365267931e-11	amiD
4	ENSB:-PtSFJ_OR6krjw7	1680.955	-3.82309	1.2187	2.01798969093827e-05	0.000568	spaP_1
5	ENSB:-rzC9Vl4onOI7le	123.6928	-10.244	1.325928	2.44073089059654e-15	2.71409275034336e-13	ybiR
6	ENSB:_-2vVeEECegl2FM	478.7796	1.426749	0.755788	0.001231	0.018821	rocR
7	ENSB:_0H9s0QfmKwbzYI	1467.277	-8.63672	1.341967	3.39634139597456e-11	1.96193851029803e-09	ybhF
8	ENSB:_6ILvhm6FxWGoLQ	486.6085	3.414803	1.197763	5.43350273272692e-05	0.001381	mgtC
9	ENSB:0czyN4GlHP_OO-_	810.6138	1.597727	0.80102	0.000964	0.015612	trxC
10	ENSB:0Dc1DztET3sCeWq	11.02384	-6.42561	1.926083	1.56573536768202e-05	0.000458	cutD
11	ENSB:0GI85A9sb3Nvhe-	3823.361	2.609963	0.413219	3.85415161106271e-11	2.19785466230858e-09	osmB
12	ENSB:1Is14-u1ODQdcnW	3354.464	-2.88278	0.632844	3.50431385485601e-07	1.4300172501284e-05	tar

13	ENSB:1MgII4EIB1BOk5Y	554.4387	2.209094	1.539895	0.001941	0.026327	sptP
14	ENSB:1N_f0Oj4_tvI4A6	710.7923	-9.78863	0.958807	4.74310687769534e-25	2.3441488213321e-22	ltaE
15	ENSB:1P8P8pq-pVcar4Q	564.9394	-9.11388	1.09448	2.49802404165888e-17	3.83145204734438e-15	ydcV_1
16	ENSB:1sONqydI17ceP49	34.62714	2.285917	1.508692	0.001595	0.023109	fumB_1
17	ENSB:27_IRIYOS3Dvc2l	2376.098	-2.43376	0.393866	1.28904475992942e-10	6.5904265427196e-09	lsrA
18	ENSB:2EHxa2rJTjN0sd8	52.6207	-9.03669	1.374103	1.30279146336439e-11	7.83083301222272e-10	ybjO
19	ENSB:2N5JtakXaRWhzmJ	518.5421	-10.095	0.976879	1.19580714672374e-25	6.648687735784e-23	clsB
20	ENSB:33PtdMsBENhzNhy	475.556	2.385269	0.677268	2.59632947150147e-05	0.000708	umuC_1
21	ENSB:3G3yEY0iMtF6npP	450.9836	-9.48419	0.951224	5.83725271386365e-24	2.36037273375141e-21	rimO
22	ENSB:3OTmon_bfKVhoA7	17661.23	5.304843	0.779633	2.733238001804e-13	2.06058349695325e-11	deoC
23	ENSB:3p6w2dWVysCc06H	8039.676	2.424211	0.924452	0.000192	0.004125	lpxP
24	ENSB:4_5fmaAyZNwJKpn	11932.74	2.621537	0.676045	7.24823940036053e-06	0.000225	degP
25	ENSB:4s_rpOlb3q36EfE	113.4426	2.134816	1.365365	0.001636	0.023478	yjiK_1
26	ENSB:4u-pPhc2GQ8FYui	64.27398	1.567217	1.155789	0.003706	0.043938	yicJ_2
27	ENSB:4VrzItBD0riQGow	3716.129	-2.50004	1.103518	0.000359	0.006845	ssaV
28	ENSB:4YsPouWoEy7GMVq	52931.58	3.00177	1.012801	5.73633602298246e-05	0.001425	recA
29	ENSB:59zeTns-6eB2_8V	270.8879	2.594037	1.022173	0.000199	0.004223	manC1
30	ENSB:5DfaT9NTSQ0SLhO	9745.915	1.778139	0.43841	6.05240051302371e-06	0.000191	yccA
31	ENSB:5l5e-DXX5a_cqUf	169.0008	-3.15334	0.749473	9.44141998916588e-07	3.44224886162376e-05	arcC1
32	ENSB:5ztfu2-Uumdc6kz	1069.073	-2.03169	1.144651	0.001216	0.018715	tsr_1
33	ENSB:65XyhnSNckJwBut	385.8486	-10.1246	1.179856	2.19007652128464e-18	4.23541755072785e-16	gsiA
34	ENSB:6HT8_X-lYZKoRvn	1265.14	-2.22747	1.048186	0.000564	0.009951	otnC
35	ENSB:6hv5Fn9YDGl3paf	70.08054	2.292448	1.488477	0.001519	0.02223	fimF_2
36	ENSB:6iTojDEIqvJlORV	60.60706	-9.26221	1.329133	8.67858716602593e-13	6.12735804991799e-11	pflD
37	ENSB:6JKNlV14siPFREv	363.2742	-2.16342	1.535821	0.0021	0.027884	cesT

38	ENSB:6S41-8eI6gYwz9r	168.0919	-8.02558	1.059538	1.20022533521561e-14	1.18635606467534e-12	ybjN
39	ENSB:6yGfAFTBRpzkoQV	1670.796	-9.36686	1.002089	2.61283624680298e-21	7.26368476611229e-19	ybiT
40	ENSB:7--dk5KD0whwTRF	73.41644	-1.95618	1.295337	0.00206	0.027521	cheY
41	ENSB:7baztSxcsBQlhw9	186.819	-9.14917	1.135746	2.3428912549038e-16	2.97748008623203e-14	gstB
42	ENSB:7VtS2GUbuPgj09a	2717.283	1.196846	0.865328	0.004356	0.049687	ycjX
43	ENSB:8dQ4Z60lH41SKb0	411.9174	-4.16478	1.979657	0.000233	0.00483	spiC
44	ENSB:8Sgdr2lQsdqjk2J	173.7279	2.195325	1.214645	0.00102	0.016088	higA
45	ENSB:9AOBn1XQJwhvIrD	1893.025	-9.45003	1.149758	5.68789140644008e-17	8.16120676640176e-15	poxB
46	ENSB:9jLRwq38k7qBEoB	210.6621	3.212362	1.303713	0.000148	0.003284	malK
47	ENSB:A02JaVhbgpOyuBq	271.1157	-10.4591	1.255999	1.73570625778639e-17	2.7572933695121e-15	ybjG
48	ENSB:A6XwzlrFHQ3m_G4	433.1369	-9.01953	1.069308	1.02426656851624e-17	1.68738433213342e-15	macA
49	ENSB:AHe6_O0RECnZNcW	1261.081	-3.79023	0.840268	9.52374752897905e-08	4.19422069395038e-06	cheR
50	ENSB:ahmOlSaf4Vrmq8z	1150.639	-2.16566	0.707215	8.95746316378755e-05	0.002075	flgK
51	ENSB:aKJsTM3SSjZbR9i	320.1099	1.670884	1.15165	0.002859	0.036329	vapC_2
52	ENSB:aOSQbPTxCMDOKTZ	1250.366	4.130622	1.33778	2.10847452071755e-05	0.000586	rtcB_2
53	ENSB:Auzl1y-whOEYFXk	224.692	1.033751	0.650522	0.002554	0.033408	fdnI
54	ENSB:b9KMbh7Yx4Wsl6x	93.75196	3.026986	2.423571	0.00186	0.02569	pphB
55	ENSB:b9LD6IQec-ZTb5Q	441.1036	2.598043	0.412536	4.44562577612471e-11	2.47176793152534e-09	cadB
56	ENSB:BgRQukQgiCP4lXV	126.8932	3.132969	1.140095	8.42464038267692e-05	0.001983	algC
57	ENSB:BnoXswoiLKmd9kr	366.0101	-9.39643	1.264555	2.94016842913552e-14	2.6689528924071e-12	potF
58	ENSB:bo4_i6R88PLTOiR	286.7694	1.415983	0.743932	0.001185	0.018436	livJ
59	ENSB:BQ1R1qkDDo5-N1w	101.4523	1.997228	0.922433	0.000601	0.010573	ansB_2
60	ENSB:BRkUBIka5X1-yO8	84.03147	1.82967	0.658408	0.000177	0.003853	cysE_1
61	ENSB:BtvVN3CReZXc877	1757.596	-4.7977	1.315611	3.44251156680537e-06	0.000117	pipB
62	ENSB:BZ9930kd4rQPzyJ	320.8841	1.982198	0.97848	0.00079	0.01347	fcl

63	ENSB:C_22vfkLGFHGhvJ	1395.792	2.447294	0.604404	5.05855836922764e-06	0.000161	pstC
64	ENSB:c15JaAOmd8p6qxQ	940.9681	-9.38079	0.861109	3.63641321877528e-28	3.23495319942249e-25	dacC
65	ENSB:cEXMJOr1ZsnLMTc	92.26456	3.223485	1.2743	0.000128	0.002895	klcA
66	ENSB:chthiH2Z3m21vfi	75.36256	-1.7309	0.848237	0.000854	0.014385	pdeH
67	ENSB:cjkXFi9KNotiz7M	588.7698	-1.88228	0.547877	3.77973915154746e-05	0.000989	vanX
68	ENSB:cV-NGO2Az_MDdBp	1100.895	-8.83276	1.24924	4.56200533989168e-13	3.27287092771584e-11	aspT_1
69	ENSB:cX1A7dJjh45DdTV	227.5241	-8.48657	1.003855	9.62056808427681e-18	1.64585718611013e-15	glnP
70	ENSB:D_HJcPQC__hNWfq	3278.999	1.189325	0.859209	0.004357	0.049687	yebE
71	ENSB:D9Sr-IvgdbDFaU2	396.9276	2.467274	1.314959	0.000742	0.01269	sbp
72	ENSB:Db4IkZxO5PNKX5t	4676.202	-2.0899	1.375393	0.001828	0.02549	otnK
73	ENSB:deBnTPWRjiu-6ed	64.25236	-9.29121	1.398765	8.04588859918704e-12	4.90248116290191e-10	rlmF
74	ENSB:dgBQXUmVBaKm_kk	143.4327	1.722495	1.208575	0.002882	0.036447	wzxC
75	ENSB:DIOT_VYuU3Gkac-	692.7045	-10.8167	1.313939	3.44006686181354e-17	5.10047246711554e-15	ybiS
76	ENSB:dtWg3NDlKzvW3W5	172.9144	2.820877	1.117497	0.000171	0.003756	nupG
77	ENSB:Du6TdiGezQzmB0h	172.413	-2.20845	0.821298	0.000194	0.004125	argF
78	ENSB:DuXz8EXPLcbA9TH	37.4636	-7.63014	1.397188	8.36785366205515e-09	3.95959713710865e-07	opgE
79	ENSB:DyRdc7xUnnYcieK	972.2421	-2.9414	0.675907	7.57216697467149e-07	2.79816810844928e-05	motA
80	ENSB:DyTbKo3rCbY1BlH	5301.863	2.782895	0.935816	6.85452219345203e-05	0.001657	yebF
81	ENSB:e0ECCumw_YjzJpm	210.1584	-3.85289	1.400678	5.63119583598983e-05	0.001407	dlgD
82	ENSB:Eantr_Hpc0uJdJs	135.7873	2.181638	1.182268	0.000963	0.015612	epsJ
83	ENSB:ELzmd5bPhHwagMG	2228.495	6.197241	0.489197	2.16050852210237e-37	4.80497095315567e-34	narK
84	ENSB:eojTGJ3UKW-u-nN	856.9387	1.499491	0.870212	0.001784	0.025038	moaA
85	ENSB:EONMjdgX_mo0RTG	785.7116	1.768368	0.98335	0.001373	0.020557	mprA
86	ENSB:EozdqgMgZF8A3JS	10978.92	-9.33577	1.229165	8.55171170741917e-15	8.64500310786374e-13	clpA
87	ENSB:f_592G8SxL4Ck4u	616.9671	1.72112	0.71772	0.000401	0.007437	pspC

88	ENSB:F140mCeTPlsRvpU	707.235	-4.99262	1.833965	5.53835578670811e-05	0.001397	spaR_1
89	ENSB:FEf_BjW1eaHWm1P	44.90987	-7.07129	1.251116	2.38971882009690e-09	1.15537709910772e-07	gsiB
90	ENSB:FoNVxLS1bCqbtaq	219.6672	2.34738	1.125006	0.000555	0.009883	recX
91	ENSB:fsu4kdMTOtLJ7lk	740.497	2.918217	1.037995	8.7089688398222e-05	0.002028	marA_1
92	ENSB:fWeFBBmuIlU5Okd	961.655	1.614656	0.957318	0.001852	0.025689	dinF
93	ENSB:fXmGzXCm0R0ut_4	415.4914	-1.26974	0.646535	0.000979	0.015779	yedK_2
94	ENSB:G-gXELAoJCge3Xj	938.4933	-8.88809	1.186466	2.06929151199228e-14	2.00091492290036e-12	ybhQ
95	ENSB:G8eMT41UI2B_Cjs	7953.702	1.393461	0.699479	0.000954	0.015593	ahpC_1
96	ENSB:gD4Npc9GB0esCQW	71.90916	-1.57992	0.743528	0.000723	0.012418	fliZ
97	ENSB:GEZVUUYEtTJGuOP	2957.445	2.873985	0.652106	6.78801378217823e-07	2.53723405908645e-05	eco
98	ENSB:GI9Sn7mVbDWJu5G	134.7811	-9.48028	1.274447	2.71191804292289e-14	2.51304405310854e-12	mdfA
99	ENSB:GQqOsPhhj08sqIL	523.297	2.450535	0.514091	2.90009191614884e-07	1.20557091990935e-05	yaiY
100	ENSB:gz7PNYHiztsYEJR	2658.717	-2.12267	0.579062	2.04085312708284e-05	0.000571	lsrK
101	ENSB:H3Ux-giuHfQlSw4	9.967493	2.709196	2.850547	0.004008	0.046834	ddrA_2
102	ENSB:H97V9nztmJ1Aa0y	658.6147	-1.3369	0.851129	0.002638	0.034213	lsrR
103	ENSB:HaLOa6LoyZplUhW	290.7806	-9.0808	1.252051	1.17492814936022e-13	9.86052907236654e-12	grxA
104	ENSB:HbcBV9usN-bnq33	227.5214	1.192682	0.534191	0.000449	0.008214	yfbR
105	ENSB:HG9PGCdBffjtkaq	1066.828	-8.93141	1.204388	3.62089036030387e-14	3.15798437698659e-12	macB
106	ENSB:HgeZW731YKVrVak	11852.08	1.838063	0.601805	9.48079624019768e-05	0.002185	ilvC
107	ENSB:hqyJMoM1aEe1Dar	90.07394	1.848057	0.710184	0.000259	0.005293	ydcO
108	ENSB:I_IET4mRwAuXtq4	61.90272	2.020211	1.220527	0.001519	0.02223	assT_1
109	ENSB:I122ZNcwDYGqamR	599.5632	-9.88512	0.951758	7.35281213330061e-26	4.67218690984587e-23	ybhS
110	ENSB:I5OYuOtwabIl-Tv	623.209	-2.01799	0.705555	0.000146	0.003268	rnc
111	ENSB:idfjmhvTOxfGFia	394.7413	-8.15785	1.149782	4.05318149451672e-13	2.95550021108366e-11	ybhN
112	ENSB:IdZW2FtwtfKSYZG	86.94241	2.998928	1.07722	8.64554971815166e-05	0.002024	prfB_1

113	ENSB:IE7FRkR4IFt-sXk	181.2137	-8.16347	1.007437	1.86013546043645e-16	2.58558829000666e-14	ybjP
114	ENSB:IiVQdMZADQ80hJT	655.437	5.122788	1.397699	3.6287659519714e-06	0.000122	dinI_3
115	ENSB:IlJm5nil-0hgz46	312.7382	1.826826	0.906776	0.000871	0.014493	deoR_2
116	ENSB:imjOr6AzjOvAIja	129.8931	-1.28986	0.767126	0.002024	0.027199	lsrG
117	ENSB:iohL1ZpYZhgUePS	229.0503	-10.2015	1.29015	5.89566862450502e-16	6.90103527415745e-14	hcr
118	ENSB:is1yxMsXViwf4zD	312.8388	2.805891	1.58397	0.000723	0.012418	higB
119	ENSB:iu98y189zXQ-gjD	164.9084	2.479124	0.505318	1.44668679724448e-07	6.2474396836344e-06	pspD
120	ENSB:IxLU7fwcbcANTjt	657.6604	1.837543	0.623821	0.000122	0.002777	phoU
121	ENSB:iYZ9ooMExDZ-DUs	4824.252	-3.09259	0.806135	4.27729865121069e-06	0.00014	sspH2
122	ENSB:JdEfxpmf9mQLmye	1830.708	-1.51209	0.451245	3.44827158831674e-05	0.000908	ybdL
123	ENSB:JK-Y6v6queU7QuN	1029.816	-2.20751	1.242572	0.001067	0.016776	otnI
124	ENSB:jMQpBT4rrBcep1k	371.9535	3.078355	0.492363	1.95576776555469e-11	1.15990066949163e-09	ycaC_2
125	ENSB:JvYN5OWNLJkArhY	2360.633	2.441749	0.793059	7.29443193002032e-05	0.001744	fkpA
126	ENSB:k-vrn_BQdTeLfjl	1873.351	-1.27135	0.869684	0.003495	0.04224	rbsC
127	ENSB:k9gr79ouyqZuXLm	383.9811	-9.97721	1.34936	3.33289609637339e-14	2.96494436733377e-12	potA_1
128	ENSB:KbkB1ItJOSVgHVN	1992.815	-8.14175	1.500106	1.12390924880639e-08	5.2074461861363e-07	artP
129	ENSB:KFB13d4Ed8g6K74	1353.31	1.879237	1.261097	0.002267	0.02983	napG
130	ENSB:KhQe-Ns6ciWxQrd	856.1325	-8.91629	1.154363	3.45158003466378e-15	3.57037860329872e-13	ybiO
131	ENSB:kkStrG1s9rRp_Bf	4872.348	-2.16256	1.533355	0.002095	0.027884	ltnD
132	ENSB:kmYOdGiIAyhLIXY	252.6573	2.059795	1.334508	0.001794	0.025092	allS_1
133	ENSB:KWSQ7Yp4fBcMzfH	79.52383	-8.68155	1.379351	8.30126524227631e-11	4.44867804790904e-09	ybiI
134	ENSB:KZzimdAzmzh_XfA	916.6591	1.750898	0.417229	3.7294897928658e-06	0.000124	nfsB
135	ENSB:L0dIBolAByiZfsM	187.9652	-9.9467	1.26438	8.74873041310111e-16	9.97803919935225e-14	iaaA
136	ENSB:L5SCILr8025oaFp	506.9963	6.30651	1.438785	5.07593811770042e-07	2.01587256674388e-05	dinI_4
137	ENSB:lFtY6RUkylydLLk	164.5306	-8.95811	1.15406	2.5389827543859e-15	2.75448665646548e-13	nfsA

138	ENSB:LjGHhtGBtV1WKoN	3348.847	-2.57415	0.851843	7.43086612244026e-05	0.001768	cheA
139	ENSB:LkXV1lwIgHvuMHK	1182.96	-1.82839	0.888262	0.000798	0.013552	lsrE
140	ENSB:Lp3OIaJkTpJ6Sx8	6581.107	3.424208	1.04039	1.66851257539747e-05	0.000485	rtcA
141	ENSB:lPsafB7EagG_TxH	328.577	-9.70676	1.410025	1.41544062203743e-12	9.68596905665001e-11	potH
142	ENSB:LwE4ZMFTWv9sHtd	237.3541	2.766602	1.10594	0.000187	0.004038	mtlK
143	ENSB:Lwtc6S5xYrTtXtc	809.9385	-2.48453	0.396048	6.47873847461496e-11	3.51432057744968e-09	lsrD
144	ENSB:LxNLsyWmTvPa-xj	1776.968	1.471167	0.687421	0.000684	0.011889	ndh
145	ENSB:lXZ2zj3Ugg8zAhj	1761.574	-3.64965	0.968128	2.75209822837902e-06	9.56354134361709e-05	sctC_1
146	ENSB:LY_A5TvqHG5Uvcn	412.0308	6.028688	1.397353	5.48300915409774e-07	2.13933550152866e-05	marR
147	ENSB:LyD8jd4oOabnENB	16189.76	-8.75792	1.348265	2.28708733910624e-11	1.33854795846639e-09	ompX
148	ENSB:M6X3pTGQHMXZhzM	1149.279	1.927416	0.459044	4.02546588489365e-06	0.000133	pstA
149	ENSB:MopRnXg2sCsccJS	165.2525	2.416175	1.128762	0.000479	0.008631	wcaJ
150	ENSB:msJmPHET60FjQvv	110.2672	-7.44937	1.027662	1.24546244531148e-13	1.02589202902693e-11	oleD
151	ENSB:MtMQ_HZMIHOs05a	12.40014	-6.53973	1.997117	1.87063306925101e-05	0.00053	etfA
152	ENSB:mWTjWiTF7-s1mqW	2331.109	4.123061	0.59322	2.69928791677044e-14	2.51304405310854e-12	narH
153	ENSB:MWUSC61v119382k	256.3391	1.048985	0.69936	0.003291	0.04022	zapC
154	ENSB:N--n6745fwTgQH1	3152.684	-8.90881	1.118328	5.08006363119692e-16	6.10706027880106e-14	dps
155	ENSB:N4CsT2nO01wKY84	91.22726	-8.0376	1.293851	1.3583985081634e-10	6.86608700489865e-09	deoR_1
156	ENSB:n9lOqGt_DcewGGz	57.59334	-8.27424	1.321834	1.03401940622687e-10	5.41096272811428e-09	moeB
157	ENSB:nemc3eIJM2cUQuA	1894.098	-2.58766	1.080673	0.000264	0.005334	ssaN
158	ENSB:nQr1M5zhYPH7G93	69.79233	-9.46612	1.315044	1.60837818114006e-13	1.27751181244839e-11	hcp
159	ENSB:NwZha1hhKjMvyII	43.77497	1.513816	1.168554	0.004344	0.049687	dpaL
160	ENSB:nX2hHiGqR5Rlaoc	77.42112	2.528528	1.199047	0.000468	0.008458	gltF_2
161	ENSB:Nyoh-PKDsd7Lie9	383.6113	-2.20385	1.793774	0.002824	0.036096	sseA_1
162	ENSB:o0Iu3_IZLd1jsBE	69.08867	3.656325	1.70534	0.000238	0.004907	yhdJ_1

163	ENSB:O1ADaweldFiVBss	4662.565	3.842159	0.731131	1.79770593993334e-09	8.78702859431154e-08	pstS
164	ENSB:OAZ1wPIAAK5gjjR	43.45871	-6.35023	1.318708	8.46467689393849e-08	3.8031194771958e-06	rlmC
165	ENSB:oJLy4LvnaFsE1w2	893.82	-8.97621	0.928151	1.28455044678675e-22	4.39513875946729e-20	moeA
166	ENSB:Okl_BOuLNyjxppx	13293.44	4.599414	1.039054	1.18705840678251e-07	5.17650567977313e-06	rsr
167	ENSB:On0NzEPU_WNNzEa	31.51709	1.611329	1.124414	0.00309	0.038393	srlA_2
168	ENSB:oNf6yoYan8i6K1e	647.8173	1.909939	0.446636	3.06815798643569e-06	0.000106	cysZ
169	ENSB:OQKhjGpmRkVAO7f	142.6297	1.824781	1.162488	0.002046	0.027416	dinJ
170	ENSB:Or8T4UwHXsn_QGK	633.9024	-10.3122	1.059528	4.92005906807599e-23	1.82370189456683e-20	artI
171	ENSB:oSiJaoiDacGHcKI	624.3083	-2.75589	1.649087	0.000896	0.014816	sseI
172	ENSB:ouHmHAn4MsI-faq	91.81991	1.613553	0.90328	0.001504	0.022148	hoxN
173	ENSB:oUTnB1p74Ar_xDD	1248.025	-1.06489	0.575029	0.001204	0.018594	iclR
174	ENSB:P_Qjl7thJsYrjNx	213.4786	-7.71737	1.140493	3.75234639065389e-12	2.4911099620341e-10	clpS
175	ENSB:p2BvtIvh-O1vzCS	837.8014	-8.70351	1.024906	6.70220973449947e-18	1.2421428707939e-15	glnH
176	ENSB:pfMTyvnLKQ7LjLO	467.7581	2.002734	0.816594	0.000342	0.006552	emrB
177	ENSB:Ph2SiMqMJXzlzIy	33412.99	1.942987	0.974321	0.000858	0.014394	ompC_2
178	ENSB:pHdUNphN_TEuOtT	67.06822	-9.38626	1.350975	9.78429347916608e-13	6.80008396802043e-11	gsiD
179	ENSB:PLfzuFjTEtV-MMq	1710.647	2.586824	1.061373	0.000243	0.004989	phoR
180	ENSB:PolcH0rhp18g18m	348.7425	-10.0196	1.135373	2.64629258445224e-19	5.60509972173503e-17	mcbA
181	ENSB:POXuPJn_DDMZKtr	1161.921	-9.81972	0.913196	1.50135547969378e-27	1.11300486227966e-24	ybiB
182	ENSB:PqN_ZFXeU-OFPCi	219.928	-2.0699	0.99439	0.000676	0.011798	arcA_1
183	ENSB:Prz0pHEMfEOBR6W	425.4398	-8.51388	1.052976	2.06778409570382e-16	2.70514813461488e-14	cecR
184	ENSB:PTM4nMvK5Q2stXd	4249.642	3.662266	1.05159	7.56073114057305e-06	0.000233	tisB
185	ENSB:pugVuNbPrX3zDIu	380.0684	3.555288	0.653079	9.188613668463e-10	4.54121706636927e-08	narJ
186	ENSB:PyntiH9oQ2OYSey	2665.861	-2.65215	0.84611	5.35906161967468e-05	0.00137	bsmA
187	ENSB:qbev8lXVdFmugFf	19848.73	4.495827	0.595308	3.34558408887041e-16	4.13365500758211e-14	deoA

188	ENSB:QmsOCnCmBY-lE15	188.208	4.492171	1.120502	7.61192313674376e-07	2.79816810844928e-05	phnS
189	ENSB:QOSSRhDP1nnq7AJ	561.1965	-3.17882	0.845011	4.90460188384591e-06	0.000157	motB
190	ENSB:QRd_yclV2MNjmET	303.0135	-1.07861	0.622388	0.001705	0.024225	rhaS_5
191	ENSB:QrLKRYxkoYPKRiz	84.3781	-9.68686	1.36827	3.58655624412442e-13	2.65883369564423e-11	rcdA
192	ENSB:qSe0kIy4FJpHKue	158.7711	1.335179	0.783644	0.001913	0.026261	malG
193	ENSB:qVCF61QwrCy0gdI	1203.22	-1.623	0.840187	0.001101	0.017237	denD
194	ENSB:qvNrsjSLs3pw5qS	251.9361	2.080105	0.430074	3.16238932542325e-07	1.30243589995209e-05	fucP_2
195	ENSB:rAjBWhw4iYNc-Rp	67.42933	1.533479	0.775555	0.001005	0.016024	ftnA_2
196	ENSB:RCJfnfqdBSGhdY8	226.024	2.823198	0.862682	3.22660329878608e-05	0.000854	uhpT
197	ENSB:RiaojE_DmQoyy0i	98.86506	1.382848	0.888429	0.002693	0.034719	dkgB
198	ENSB:rTMQVKGxlpxBMjy	465.5204	1.156574	0.800139	0.003759	0.044347	hslJ
199	ENSB:RWa83qOBFvHPH7v	59.53652	-7.49027	1.227785	2.33793953344276e-10	1.16844438705095e-08	rhtA
200	ENSB:s9NhUH2saFhZCvk	645.3653	-1.41944	1.004032	0.003648	0.043735	yihI
201	ENSB:SbjLADvyavUUhWQ	204.7177	1.06574	0.726591	0.003605	0.043339	nlhH
202	ENSB:sBlQnjwhLaBZqLq	114.6283	-8.39189	1.243786	4.46563535824515e-12	2.921050893158e-10	ybjI
203	ENSB:sbup8jTtu0dT_cw	10.69301	-6.40694	1.894998	1.38873686574831e-05	0.000409	ulaA_1
204	ENSB:SD-f6ClT8OOqdtZ	2012.57	-1.62612	0.821147	0.000991	0.015854	rcsC_1
205	ENSB:sGDfH7eSOJKacTr	369.46	2.010469	0.915136	0.000562	0.009951	yiaD
206	ENSB:SGrbwo-oUEm4zvJ	123.5943	1.767568	1.090809	0.001937	0.026327	panE_2
207	ENSB:sHD0lu3QiXfTGV1	162.0276	1.424935	0.922157	0.002722	0.034988	malF
208	ENSB:SotN5UG-Zr-Elxa	1593.013	1.401517	0.777231	0.001503	0.022148	lexA
209	ENSB:ssBXywvH3FkE39h	492.3895	-2.06548	1.668575	0.003052	0.038216	lpxM
210	ENSB:SsyqOJ2HiAseg_7	414.7952	-1.12882	0.749523	0.003215	0.039506	fljB
211	ENSB:T4qTpJriW7Aa49f	2415.287	-3.04949	1.424225	0.000312	0.006058	sopD2
212	ENSB:TlFhO6LXUEuWoHD	62.97644	-6.52208	1.221328	8.07289966832976e-09	3.86110298115385e-07	lysO

213	ENSB:TmqdudWXiXyYuyV	1496.338	-3.07912	0.784786	3.20024376938214e-06	0.000109	cheB
214	ENSB:TqQJ_rPNWjzhFZP	327.2901	2.230948	1.028269	0.000518	0.00926	napH
215	ENSB:TtkX8Maanu18MZm	519.4232	-9.01012	1.065892	8.7885474265334e-18	1.56365835812882e-15	ybjJ
216	ENSB:ttQLVR_ZC97BsRm	4908.673	4.913576	1.135548	2.22561903932292e-07	9.33920140274374e-06	recN
217	ENSB:TUqJT_twiaNXwwb	294.2398	1.078525	0.638695	0.001934	0.026327	moaB
218	ENSB:Tv02f9pncsN10FC	1053.667	-1.58627	1.037662	0.0026	0.033911	lsrB
219	ENSB:TV57IWpO_5Dwuqf	51.28825	4.074952	2.16911	0.000383	0.007179	yehA
220	ENSB:TVKda-ubWqV59vm	3234.176	-1.3908	0.373831	9.17175323181667e-06	0.000279	metR
221	ENSB:TWZWjzluPt_Gv1o	570.1307	-8.80741	0.924984	5.66232834260803e-22	1.67906909786137e-19	artJ_1
222	ENSB:TYqgHZJV5y72_rh	305.2177	-2.0593	1.754548	0.003443	0.041849	yscU
223	ENSB:u-rbf0S4fcMXGin	27200.35	1.692024	0.586812	0.000134	0.003023	rpoH
224	ENSB:u_uT_77uH97_-hZ	4277.984	4.627931	1.031314	8.97414471496906e-08	3.99169956921824e-06	spy
225	ENSB:u0PMNiNjPTtKZbL	1028.773	3.235252	1.028344	2.95355688258313e-05	0.000791	mgtB
226	ENSB:udprT8xzAtlYy_S	1071.242	-4.27495	1.148055	2.4171430371422e-06	8.4657104166996e-05	sseL
227	ENSB:ujjrhnxoKVPaN1S	159.9208	2.988101	1.456484	0.000384	0.007179	dsbA_1
228	ENSB:umpyRMOscJGm1Pc	709.4647	-9.57966	0.861986	3.02575934860202e-29	3.36464439564545e-26	artQ
229	ENSB:UQdqLKvQwV-7rPF	293.0917	-1.34932	0.729188	0.001336	0.020144	ogt
230	ENSB:USeZUI0sGyiDNRD	84.01716	-9.62106	1.448247	7.45874693813373e-12	4.67274737758012e-10	rimK
231	ENSB:USNovuHkmBptQTO	75.81553	3.530904	1.881033	0.000432	0.007969	marA_2
232	ENSB:UWKxK0u0-TgAH1s	133.7098	-1.13599	0.763597	0.003375	0.041124	yhdE
233	ENSB:uX1WuTbOVyiC76f	575.6339	-2.46322	0.979979	0.000231	0.004801	yhcO
234	ENSB:uxAbdXuA1zUsAZq	1230.498	-1.25196	0.657938	0.00114	0.017788	pgpC
235	ENSB:UzkyusjLLcFPTRa	131.2726	4.578812	1.179291	1.31003300653816e-06	4.6616214504654e-05	ompN_1
236	ENSB:vbQF1He7lPkCrAx	2712.35	2.891389	0.784527	9.3232742689993e-06	0.00028	sulA
237	ENSB:vF6gRy3g9f0Ym7X	357.7734	-10.0607	1.128564	1.17097061282301e-19	2.74130383465092e-17	artM_1

238	ENSB:vIYkfot3Ed63RX2	297.8796	1.496417	0.88703	0.001935	0.026327	malE
239	ENSB:VKfz_NJLO2Hb5Xc	208.0613	-1.91477	0.785785	0.000363	0.006894	flgM
240	ENSB:vKx6jnM1QQqfIFi	131.0631	-9.34774	1.40591	7.62127182620577e-12	4.70825237263378e-10	ybjM
241	ENSB:vmyaUPwU6eECxsH	6129.466	-2.76606	1.100087	0.000182	0.003958	pipB2
242	ENSB:VtOooXgDNeCVhQ-	149.3481	-8.83192	1.14232	3.30428823202724e-15	3.49939858477552e-13	gsiC
243	ENSB:VvT3nIokh-_okZK	980.0452	-2.40346	0.68006	2.47692668789809e-05	0.000684	nreC
244	ENSB:w8dubPxJXBvBpGj	589.4394	-8.38888	1.168074	2.16287436915007e-13	1.68780091122447e-11	dinG
245	ENSB:wA2muBl2LYBX9CG	151.8962	3.222368	2.023199	0.000852	0.014385	yafQ
246	ENSB:WbL5A_r4uEC3Q4m	1467.791	3.720316	0.95854	1.6761394354912e-06	5.91703826116259e-05	narI
247	ENSB:WBT6GcA3diy2jhH	254.7218	-10.2284	1.4407	2.6841895755676e-13	2.05849572967667e-11	ybiV_2
248	ENSB:wMIXprl2RI_8KNm	952.1589	-1.86334	0.731559	0.000291	0.005783	lsrF
249	ENSB:x-qfZa5JzYtaIV_	86.7629	-8.0521	1.175216	2.19780578920261e-12	1.4811879015717e-10	rhlE
250	ENSB:x1QxJfXB8V9pfE6	73.01095	3.060333	1.455682	0.000334	0.006467	lpfA
251	ENSB:X2aTfEDKGTQXKXY	531.2002	-9.7109	0.957139	9.28901513236189e-25	4.13175393087457e-22	ybhR
252	ENSB:x326ncGZF6szjxF	233.8995	-8.07223	1.202884	5.68196906911473e-12	3.66281136513367e-10	glnQ_2
253	ENSB:x5oBmKPAwNI2qN_	32.19787	-7.31425	1.544224	2.15173835243055e-07	9.11517351582008e-06	cysL
254	ENSB:x7ojb41mc19d-5O	256.6026	-9.09893	0.984107	7.22656849729672e-21	1.89081039270446e-18	cspD
255	ENSB:XMapq7l5Jhfsam5	1947.614	-2.79404	0.428935	7.07918829574609e-12	4.4983185056398e-10	racX
256	ENSB:XMYYsW3XyIhK4sH	337.9925	1.536957	0.953349	0.002243	0.029603	dinB
257	ENSB:xpaDYjMXM7NzN7i	10891.44	5.483382	0.370181	9.39581707684795e-51	4.17925943578197e-47	narG
258	ENSB:xUdGN_uVtG_gPo8	1273.986	-1.62045	0.359141	1.03886572687359e-06	3.75680874238514e-05	metB
259	ENSB:YB-RwuQxzp_gcwl	498.2953	2.172023	0.586029	1.78956906681929e-05	0.00051	pstB
260	ENSB:YNlV2PsCOohH4n1	296.2253	-2.1391	1.932941	0.003706	0.043938	sseJ
261	ENSB:ys91BsMJCze1PXf	425.9663	3.191501	0.879991	7.57983254202058e-06	0.000233	wzc
262	ENSB:YX_FM0OahtKqbQv	11274.05	2.352266	0.663272	2.49399501991514e-05	0.000685	htpX

263	ENSB:z9lYmqkm3zlI1By	213.5444	-1.53939	0.841144	0.001395	0.020825	dgoD_1
264	ENSB:zdjt5EdqE5NNoMc	9.484502	3.465225	2.821554	0.001736	0.024591	torD_1
265	ENSB:ZfsgkWeWHj43APi	223.0925	1.841646	0.944904	0.000985	0.01582	mshA_2
266	ENSB:ZRfRJk4KUTmOG2P	251.6883	3.146008	0.931175	1.7042069389424e-05	0.000489	gmd
267	ENSB:zu7dC50DeGBCD4o	235.5528	5.963457	0.965638	4.33545628900517e-11	2.4410265282905e-09	ail_5
268	ENSB:zV4tantNe5cXUdP	1100.679	-2.01152	0.83539	0.000372	0.007017	lsrC
